# One Patch Is All You Need: Joint Surface Material Reconstruction and Classification from Minimal Visual Cues

**DOI:** 10.3390/s26072083

**Published:** 2026-03-27

**Authors:** Sindhuja Penchala, Gavin Money, Gabriel Marques, Samuel Wood, Jessica Kirschman, Travis Atkison, Shahram Rahimi, Noorbakhsh Amiri Golilarz

**Affiliations:** Department of Computer Science, The University of Alabama, Tuscaloosa, AL 35487, USA; gcmoney@crimson.ua.edu (G.M.); gmarques@crimson.ua.edu (G.M.); scwood4@crimson.ua.edu (S.W.); jlkirschman@crimson.ua.edu (J.K.); atkison@cs.ua.edu (T.A.); srahimi1@ua.edu (S.R.); namirigolilarz@ua.edu (N.A.G.)

**Keywords:** material reconstruction, minimal visual input, partial convolution, robotic perception, surface classification

## Abstract

Understanding material surfaces from sparse visual cues is critical for applications in robotics, simulation and material perception. However, most existing methods rely on dense or full scene observations, limiting their effectiveness in constrained or partial view environments. This gap highlights the need for models capable of inferring surfaces’ properties from extremely limited visual information. To address this challenge, we introduce SMARC, a unified model for Surface MAterial Reconstruction and Classification from minimal visual input. By giving only a single 10% contiguous patch of the image, SMARC recognizes and reconstructs the full RGB surface while simultaneously classifying the material category. Our architecture combines a Partial Convolutional U-Net with a classification head, enabling both spatial inpainting and semantic understanding under extreme observation sparsity. We compared SMARC against five models including convolutional autoencoders, Vision Transformer (ViT), Masked Autoencoder (MAE), Swin Transformer and DETR using the Touch and Go dataset of real-world surface textures. SMARC achieves the highest performance among the evaluated methods with a PSNR of 17.55 dB and a surface classification accuracy of 85.10%. These results validate the effectiveness of SMARC in relation to surface material understanding and highlight its potential for deployment in robotic perception tasks where visual access is inherently limited.

## 1. Introduction

Surface material reconstruction and categorization are essential for robotic perception and physical contact with the environment [1], and safe human–robot interaction. These abilities let robots to recognize textures, hardness and reflectivity, important clues that influence how a robot perceives, manipulates and responds to real-world surfaces [2]. Understanding whether a surface is metallic, soft or granular has a direct impact on the applied force, grip stability and tool trajectory in robotic manipulation activities like grasping, drilling and polishing [3]. However, visual inputs obtained in unstructured or congested surroundings are frequently insufficient due to occlusion, sensor noise or limited views. Such limited observations make it difficult to derive precise material properties or reconstruct surface appearance from incomplete data. As a result, there is a growing need for perception frameworks that can reason beyond limited seen pixels and infer missing information in order to accomplish accurate surface understandings and reconstruction in robotic systems [4].

Early image reconstruction methods relied heavily on convolutional architectures that are effective at modeling local spatial patterns and capturing fine-grained textural details. Classical frameworks such as Convolutional Autoencoders [5,6] and U-Net established the foundation for dense image restoration tasks including denoising, inpainting, and semantic segmentation. However, these architectures primarily rely on local receptive fields, which limits their ability to reason about global context when large portions of the image are missing or occluded [7].

Recent advances in vision transformers introduce attention mechanisms that capture long-range dependencies across image regions [8]. Models such as Vit [9], Swin [10], and MAE [11] have demonstrated strong performance in image understanding and restoration tasks under conditions where sufficient visual context is available. Nevertheless, many of these methods assume relatively dense observations or treat reconstruction and recognition as separate objectives. As a result, accurate perception from extremely sparse visual input remains a challenging problem. In robotic environments, visual observations are often limited due to occlusions, restricted viewpoints, or sensor constraints, making it difficult to reconstruct surface appearance and infer material properties from partial data. Addressing this challenge requires models that can reason beyond visible pixels and jointly learn reconstruction and semantic understanding from minimal visual cues.

To overcome these limitations, we developed SMARC, a dual-purpose framework that integrates partial convolutions and mask propagation within a U-Net like architecture [12]. Unlike prior methods, SMARC operates exclusively on valid, continuous (unmasked) pixels and dynamically updates the mask at each layer to maintain spatial consistency. This design enables the network to reconstruct missing regions with high structural fidelity while performing joint surface classification. Through this mask-aware learning strategy, SMARC bridges the gap between incomplete perception and reliable material understanding. The key contributions of this work are as follows:

- **SMARC** is proposed as a unified framework for joint surface reconstruction and classification from only 10% visible input.
- A mask-aware Partial Convolutional U-Net is designed to dynamically update visibility masks, guiding both reconstruction and classification.
- The effectiveness of SMARC is demonstrated on the Touch and Go dataset, achieving 17.55 dB PSNR and 85.10% accuracy.

The rest of paper is structured as follows. Section 2 reviews previous works related to surface reconstruction, material classification, and vision models under occlusion. Section 3 introduces the proposed SMARC architecture, detailing the Partial Convolutional U-Net backbone, classification head and training strategy. Section 4 presents experimental results on the Touch and Go dataset, comparing reconstruction and classification metrics. Finally, Section 5 concludes the paper and discusses potential directions for extending minimal view surface understanding in robotics and vision applications.

## 2. Related Works

Convolutional encoder–decoder networks have long served as a foundation for image reconstruction and inpainting tasks. The U-Net architecture introduced by Ronneberger et al. [12] established the widely adopted symmetric encoder–decoder design with skip connections, allowing the network to preserve spatial details during reconstruction. Building on this idea, Liu et al. [13] proposed partial convolutions (PConv), where convolution operations are applied only to valid pixels while automatically updating the mask at each layer. More recently, Adyapady et al. [14] demonstrated the robustness of PConv based U-Net models for restoring distorted images under various corruption scenarios. While these convolutional approaches are effective for recovering missing visual information, they are generally designed for reconstruction tasks. They do not explicitly incorporate semantic recognition, which limits their applicability in scenarios requiring both reconstruction and classification.

Transformer-based architectures have also significantly advanced visual representation learning and image restoration. Vision Transformer (ViT) [9] and Swin Transformer [15] introduced self-attention mechanisms that capture long-range dependencies across image patches, enabling strong performance in image understanding tasks. Subsequent works such as Uformer-adapted hierarchical transformer structures [16] for image restoration tasks including denoising and deblurring. Masked Autoencoders (MAE) [11] further popularized masked image modeling, where a large portion of image patches is removed and the model learns to reconstruct the missing regions during pretraining. Although these models learn powerful visual representations, reconstruction and classification are typically handled as separate objectives, and most methods operate with moderate masking ratios or largely visible inputs. Kansana et al. proposed Surformer v1 [17], a transformer-based model that combines visual and tactile cues for surface classification. Similarly, Strese et al. [18] explored multimodal feature-based approaches for material classification; however, these methods assume complete observations and do not address reconstruction from partial inputs.

Recent studies have begun exploring frameworks that integrate reconstruction and prediction tasks within a single learning pipeline. For instance, Qu et al. [19] introduced MTLSC-Diff, a diffusion-based multitask architecture designed for hyperspectral image super-resolution and land cover classification. Their results show that jointly optimizing reconstruction and classification objectives can improve performance for both tasks. Similarly, Huang et al. proposed MambaMIR [20], which reconstructs medical images from partially observed scans using masked modeling and sequence-based architectures. These works demonstrate the advantages of learning from incomplete observations; however, they mainly address moderate degradation or domain-specific imaging scenarios rather than extremely sparse visual inputs.

In contrast, the proposed SMARC framework focuses on a more challenging perception setting where only a small contiguous portion of the image is visible. Rather than treating reconstruction and recognition as separate stages, SMARC learns both objectives jointly within a single architecture. The model combines a Partial Convolution U-Net backbone with dynamic mask propagation and a multi-scale classification head trained end-to-end. This design enables the model to reconstruct surface appearance while simultaneously identifying the corresponding material class from only a 10% visible input. Such a setting is particularly relevant for robotic perception, where occlusions or limited viewpoints can restrict visual access to surfaces.

## 3. Methodology

**Figure 1:**
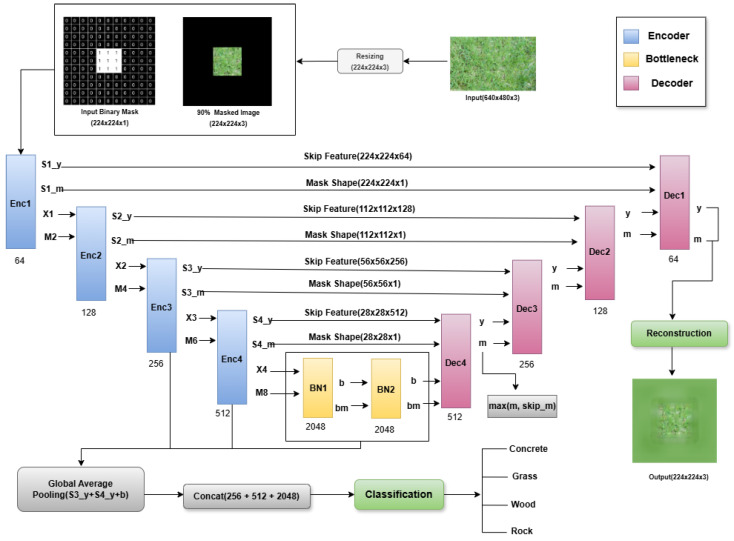
Overview of **SMARC**. The network follows an encoder–bottleneck–decoder design with partial convolutions and explicit mask propagation. Skip connections fuse encoder features into the decoder for reconstruction, while a multi-scale head pools features from S3_y, S4_y and the bottleneck b for surface classification. The model restores occluded regions in the RGB image and simultaneously predicts the material class.

We propose SMARC, a dual-purpose architecture built upon a modified U-Net framework with partial convolutions to jointly perform image inpainting and surface classification. The model is designed to restore images with missing or occluded regions while simultaneously identifying the underlying surface type such as concrete, grass, wood, or rock, supporting robust perception in autonomous manipulation and human–robot interaction scenarios. SMARC follows an encoder–bottleneck–decoder structure enhanced with skip connections, a dedicated mask propagation path and a multi-scale classification head (shown in Figure 1). Within this design, partial convolutions ensure that feature extraction and reconstruction operate exclusively on valid pixels, thereby minimizing artifacts and preserving structural integrity in masked areas. The decoder progressively restores the complete RGB image, while the classification branch fuses multi-level semantic features to enable accurate and relevant surface recognition.

### 3.1. Encoder Architecture

The encoder consists of four hierarchically arranged blocks, each designed to extract semantically rich features from partially masked input images. At each level, the encoder receives both a feature map and a corresponding binary mask as input that identify valid (unmasked) regions. This mask is propagated and refined in parallel with the feature map, enabling robust learning from incomplete observations. Each block operates using *partial convolutional layers* (PConv) and incorporates channel-wise attention via *squeeze-and-excitation (SE)* modules.

Let the input image be $X\in R^{H\times W\times 3}$ and the respective binary mask be $M\in {\left\{0,1\right\}}^{H\times W\times 1}$. The encoder processes the input through four sequential blocks $\{Enc_{1},Enc_{2},Enc_{3},$ and $Enc_{4}\}$, each composed of two partial convolution layers with kernel size 3×3, followed by ReLU activations and a squeeze-and-excitation (SE) attention module.

Each encoder block produces the following (referenced in Table 1):

- Si_y: The skip connection feature map.
- Si_m: The updated binary mask.
- Xi, Mi: The spatially downsampled features and masks for the next block.

The number of output channels increases with depth, starting with 64, 128, 256 and 512, respectively. Feature maps are downsampled using average pooling, while masks are downsampled using max pooling to preserve binary validity. By processing both features and their corresponding masks together, the encoder learns rich, multi-scale representations that remain spatially consistent and aware of missing regions. These representations provide a strong foundation for the decoder and classification branches, enabling accurate image reconstruction and reliable scene understanding.

### 3.2. Bottleneck Design

The bottleneck component operates at the model’s most reduced spatial resolution and acts as a bridge between the encoder and decoder stages. Its goal is to refine the encoded features and provide global context that can help in accurate reconstruction and classification. The bottleneck obtains its input from the deepest feature map and the binary mask output from the last encoder block, denoted $X\in R^{H\times W\times 3}$ and $M_{4}\in {\left\{0,1\right\}}^{14\times 14\times 1}$, respectively. These are passed through two bottlenecks; each bottleneck block applies two partial convolution layers with increasing dilation rates. The first block uses a dilation rate of two and the second uses a rate of four. This is followed by ReLU activations and a squeeze-and-excitation (SE) module for adaptive channel-wise attention. The use of partial convolutions ensures that all computations are based on valid (unmasked) pixels, with the binary mask being updated accordingly after each layer.

This design allows the network to increase its receptive field without further downsampling, allowing it to aggregate spatial information across a larger context. It is an essential property for tasks involving incomplete or occluded inputs. The output of the bottleneck stage includes the following:

- A feature tensor $b\in R^{14\times 14\times 2048}$, which encodes high-level semantic information.
- An updated binary mask $b_{m}\in {\left\{0,1\right\}}^{14\times 14\times 1}$, which indicates the validity of each spatial location after context aggregation.

These outputs are passed forward to both the decoder for image reconstruction and the classification head for scene recognition. By maintaining both the feature and mask paths, the bottleneck preserves structural knowledge and makes sure that the next layers work only on important areas.

### 3.3. Decoder Architecture

Thereafter, the decoder takes the high-level feature tensor $b\in R^{14\times 14\times 2048}$ and its corresponding binary mask $b_{m}\in {\left\{0,1\right\}}^{14\times 14\times 1}$ produced by the bottleneck. Its role is to progressively recover spatial details and reconstruct the complete image. Each decoding stage doubles the spatial resolution while reducing the number of channels, following a symmetric structure with the encoder. In each stage, the feature map is first upsampled using a transposed convolution with a stride of two. The upsampled feature is then concatenated with the corresponding skip feature from the encoder. This allows the model to reintroduce fine-grained details that were captured earlier. The associated masks are merged using an element-wise maximum operation to ensure that any valid region from either path remains active. The combined features and masks are refined using a pconv_block, which applies two partial convolutions with ReLU activations and a squeeze-and-excitation module for channel recalibration.

This process is repeated over four stages (dec4–dec1) with 512, 256, 128, and 64 filters respectively, restoring the spatial size from 14×14 to 224×224. Finally, a 1×1 convolution followed by a sigmoid activation produces the reconstructed RGB image $\hat{I}\in R^{224\times 224\times 3}$ (where $I\in R^{224\times 224\times 3}$ denote the ground truth RGB image and $\hat{I}\in R^{224\times 224\times 3}$ represent the reconstructed image predicted by the model.), with pixel intensities normalized to the range [0,1]. By integrating semantic context from the bottleneck with local spatial cues from the encoder, the decoder produces natural and structurally consistent reconstructions, even in areas that were initially masked or missing.

### 3.4. Multi-Scale Classification Head

In addition to image reconstruction, the network includes a classification branch that predicts the category of the input image using multi-scale feature representations. This branch supplements the decoder by utilizing hierarchical information from different encoder stages. Let the feature maps from the third and fourth encoder blocks and the bottleneck be denoted as $S3\_y\in R^{56\times 56\times 256}$, $S4\_y\in R^{28\times 28\times 512}$ and $b\in R^{14\times 14\times 2048}$ respectively. These are multi-scale characteristics from mid-level textures to high-level semantic contexts. Each of these feature maps is processed through a *Global Average Pooling* (GAP) layer to convert the spatial maps into compact feature vectors. The resulting vectors are concatenated to form a unified representation as follows:

(1) $$f_{\mathrm{cls}}=\mathrm{Concat}\left(\mathrm{GAP}(S3\_y),\;\mathrm{GAP}(S4\_y),\;\mathrm{GAP}(b)\right)$$

where GAP(·) denotes the Global Average Pooling operation that converts a spatial feature map into a compact feature vector by averaging across spatial dimensions, and Concat(·) represents the channel-wise concatenation operator used to combine multiple feature vectors into a unified representation. The resulting vector $f_{cls}$ represents the fused feature representation used for surface material classification.

The combined feature vector is passed through fully connected layers with ReLU activations and dropout regularization to improve generalization. The final dense layer employs a softmax activation to output the class probabilities corresponding to the target categories. Each classification head component consists as follows:

- Inputs: Multi-scale encoder features (S3_y, S4_y and b)
- Processing: Global pooling, concatenation and fully connected layers.
- Output: Softmax-based class probabilities for surface material prediction.

By integrating input across various dimensions, the classification head makes use of detailed spatial cues, improving the model’s ability to execute reconstruction and classification simultaneously without adding computational burden.

## 4. Experimental Analysis

To evaluate the effectiveness of the proposed SMARC model, we conducted a comparative analysis consistent with standard practices in sparse input vision research. In this evaluation, we consider five representative architectures including Convolutional Autoencoder [5], ViT [9], Masked Autoencoder [11], Swin [10] and DETR [21]. These models represent widely used convolutional and transformer-based approaches for image reconstruction and visual recognition. All models are trained and evaluated under the same minimal-view setting to ensure a consistent and fair comparison.

Although these architectures are not inherently designed to perform both reconstruction and classification under extreme input sparsity, they provide useful reference points for understanding how different model families behave when visual information is severely limited. Most recent works address either reconstruction or recognition separately and typically assume greater visual coverage. In contrast, our study focuses on a more constrained scenario where only a continuous 10% region of the image is visible. Evaluating these representative architectures under identical conditions allows us to analyze their ability to recover surface structure and retain semantic information when visual evidence is highly sparse.

### 4.1. Experimental Setup

#### 4.1.1. Data Preparation

Our training data derives from the Touch and Go dataset, which contains video examples of various textile and surface materials captured in natural environments [22]. Specifically, the dataset is acquired through paired sensing modalities, where egocentric RGB video is recorded using camera sensors while human operators probe surfaces using a GelSight tactile sensor that captures high-resolution contact information [22]. In this work, we utilize the vision-based component of the dataset, where the RGB camera acts as a vision sensor capturing the texture, reflectance, and structural patterns of materials. These sensor-acquired visual signals form the primary input to our model, making the proposed framework directly applicable to real-world robotic and embedded sensing systems that rely on camera-based perception under partial observability.

Each video was decomposed into individual frames, which were then resized to 224×224 RGB images. These images were processed through a masking script that preserved only the central 10% of pixels, simulating highly occluded conditions. The binary mask was passed in parallel with the input image and dynamically updated at each layer to track valid spatial regions, enabling the use of partial convolutions that operate exclusively on visible pixels. The dataset was partitioned into 60% (1753 images) for training, 20% (584 images) for validation and 20% (584 images) for testing, ensuring the balanced representation of all the material categories. This configuration enhances the model’s resilience to incomplete observations and supports consistent evaluation.

Each input sample consists of two components as follows:

- A masked RGB image of size 224 × 224 × 3.
- A corresponding binary mask of shape 224 × 224 × 1 that indicates which pixels are valid (one) and which are missing (zero).

#### 4.1.2. Training Strategy

The network was trained in two sequential phases to ensure stable optimization and balanced learning across the encoder, bottleneck, decoder and classification head.

**Phase A—Head Warm Up.** In the first stage, only the classification head was trained while all the encoder, decoder, bottleneck and reconstruction layers were frozen. This step allowed the classifier to adapt to high-level semantic representations without disrupting the pretrained feature extraction layers. Training was performed using the Adam optimizer with a learning rate of $2\times 10^{-4}$ for 10 epochs. To enhance robustness, class weights were computed from label frequencies to counter data imbalance, and data augmentation (random flips, rotations and brightness/contrast perturbations) was applied to improve generalization under masked conditions.

**Phase B—Fine-Tuning.** All layers were subsequently unfrozen and fine-tuned end to end with a reduced learning rate of $1\times 10^{-4}$. This phase jointly optimized reconstruction and classification objectives. The total loss combined a pixel wise Mean Absolute Error (MAE) for local fidelity, a perceptual loss for high-level texture consistency and a categorical cross entropy term for scene classification.

During training, mild overfitting was observed as training loss decreased faster than validation accuracy. This indicated that the network was memorizing training patterns rather than generalizing effectively to unseen data. To address this issue and improve robustness, several complementary strategies were introduced throughout the training process:

- **Two-Phase Optimization.** A head warm-up stage trains only the classifier for 10 epochs at $\mathrm{LR}=2\times 10^{-4}$ (encoder, bottleneck, decoder and RGB head frozen), followed by full end-to-end fine-tuning at $\mathrm{LR}=1\times 10^{-4}$ for up to 150 epochs (Adam; seed =42; and batch size =16).
- **Data Augmentation.** Rotations by $90^{\circ }$ increments (k∈{0,1,2,3}), horizontal/vertical flips, mild color jitter (brightness ±0.06, contrast [0.90,1.10], saturation [0.90,1.10]) and light Gaussian noise (σ∈[0,0.02]) are applied within the tf.data pipeline. These transforms increase the effective sample diversity per epoch while preserving mask alignment.
- **Imbalance Handling.** Inverse frequency class weights derived from the training split are applied to the classification loss to prevent bias toward majority classes.
- **Regularization.** L2 weight decay with coefficient $10^{-4}$ is applied to convolutional, transposed convolutional and dense kernels. The classification head uses dropout with rate p=0.25. Batch normalization is used throughout to stabilize activations and gradients.
- **Early Stopping and LR Scheduling.** Training employs early stopping on validation accuracy with patience =18 epochs (best weights restored). A ReduceLROnPlateau scheduler halves the learning rate after eight stagnant epochs (factor 0.5; minimum $\mathrm{LR}=10^{-6}$).
- **Multi Task Loss.** The total objective combines a mask-weighted MAE for reconstruction (weight $\lambda_{\mathrm{rgb}}=0.25$), with categorical cross entropy for classification using label smoothing ε=0.05, plus L2 regularization from layer kernels.

These strategies collectively improved generalization, reduced the variance between the training and validation performance, and ensured stable convergence across both reconstruction and classification tasks.

#### 4.1.3. Metrics

To quantitatively assess the performance of SMARC, we used a set of metrics that capture both reconstruction fidelity and classification accuracy. Following the training setup, reconstruction quality is evaluated using the Peak Signal-to-Noise Ratio, Structural Similarity Index Measure, Mean Absolute Error and Mean Squared Error, each reflecting a different aspect of pixel and structural level restoration. For the classification branch, we evaluated the accuracy, precision, recall and F1-score, averaged across all classes to account for label imbalance. Together, these metrics provide a balanced view of the model’s ability to both reconstruct missing regions and correctly identify surface materials. All the results are computed on the test set held by using the best checkpoint obtained during the training.

### 4.2. Results and Discussions

In this section, we compare the performance of SMARC to five baseline architectures: Convolutional Autoencoder, Vision Transformer (ViT), Masked Autoencoder (MAE), Swin Transformer and DETR. Our evaluation is based on three major criteria: reconstruction similarity, material classification performance and model efficiency. All the models are trained and evaluated on the Touch and Go dataset with the same minimal view configuration to ensure fair comparison.

#### 4.2.1. Reconstruction Quality

To evaluate each model’s performance to recover surface appearance from minimal visual input, we present four reconstruction metrics which are PSNR, SSIM, MSE and MAE. As seen in Table 2, SMARC has the highest PSNR (17.55 dB) and SSIM values while preserving the lowest reconstruction errors in MSE and MAE. The gains over transformer-based models and autoencoders highlight the superiority of partial convolutions in reasoning over masked spatial inputs. Notably, transformer models such as MAE and ViT did not perform well in this situation because they rely on distributed visual context, which is limited in our input regime. Although SMARC achieves the highest PSNR among all the compared models, the PSNR values remain moderate. This reflects the inherent difficulty of reconstructing complex surface textures from only 10% visible input. The relatively constrained performance across all methods highlights the challenge of modeling fine-grained material patterns under extreme sparsity.

#### 4.2.2. Classification Performance

Table 3 presents the classification performance of all the models, evaluated using four standard metrics which include accuracy, precision, recall and F1 score. SMARC achieves the highest scores across all metrics, including an overall accuracy of 85.10%, reflecting its ability to retain semantic understanding under severe visual occlusion. Although transformer-based models such as ViT and Swin demonstrate competitive classification results, their performance in reconstruction tasks is noticeably weaker, underscoring the benefit of SMARC’s joint optimization for both appearance and material recognition.

#### 4.2.3. Model Complexity and Inference Time

Table 4 highlights the model complexity and inference time. SMARC achieves the highest performance but comes with the largest parameter count and a longer inference time of 13 ms. In contrast, models like Swin and Convolutional Autoencoder are significantly more lightweight, with ViT and Convolutional Autoencoder achieving the fastest inference speed at 5.1 ms. These results underscore the trade-off between accuracy and computational efficiency, which must be considered based on deployment constraints. Among all models, the SMARC model achieved the fastest overall processing rate, handling approximately 19.1 million parameters per second. This rate is advantageous for real time robotic applications, where rapid surface perception and material classification are critical [4].

Additionally, to better understand each model’s classification behavior beyond aggregate metrics, we examine them using confusion matrices and ROC curves. These diagnostic tools offer insight into the performance of discrimination per class and the consistency of prediction with minimal input.

#### 4.2.4. Confusion Matrix

**Figure 2:**
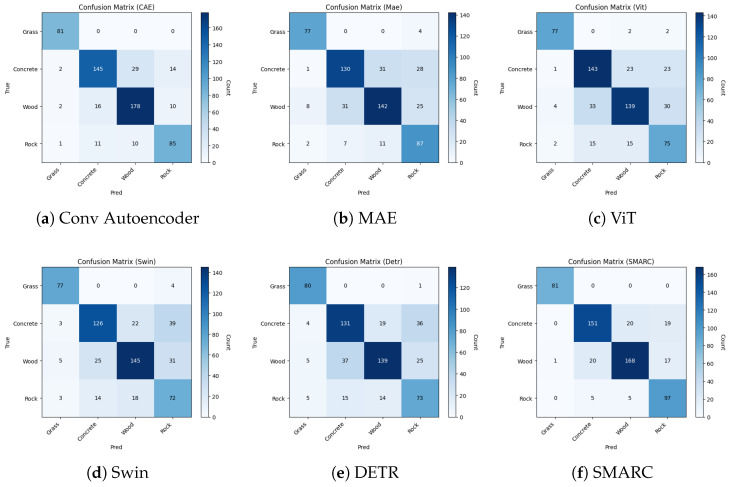
Confusion matrices of all six models evaluated on the Touch and Go dataset under the 10% visible patch setting. SMARC shows strong diagonal dominance, indicating robust class-wise discrimination.

Figure 2 illustrates the confusion matrices for all six models, offering a class-wise comparison of their classification behavior. Among all the models, SMARC (f) shows consistently strong class performance, particularly for classes like Grass and Rock, which are correctly classified with the lowest confusion. The Grass category is recognized with perfect accuracy, showing no off-diagonal errors, although Concrete and Wood exhibit some degree of misclassification, with Concrete occasionally predicted as Wood or Rock. This indicates a high level of confidence and robustness in SMARC’s predictions, even under highly occluded input. Compared to other models, its confusion matrix reflects balanced accuracy across all surface types and reduced ambiguity in decision boundaries, underscoring the strength of its joint reconstruction and classification design.

#### 4.2.5. ROC Curves

**Figure 3:**
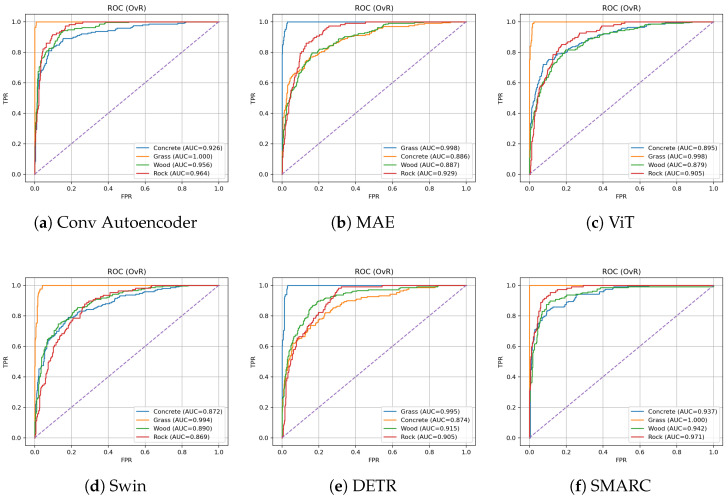
ROCcurves for all six models evaluated on the Touch and Go dataset. SMARC consistently achieves higher AUC values across material classes compared to baseline models.

Figure 3 shows the ROC curves for all six models evaluated on the Touch and Go dataset. The dashed purple lines indicate the random classifier baseline (AUC = 0.5), serving as a reference for model performance. SMARC (f) consistently achieves superior AUC scores across all material categories, with perfect classification for Grass (AUC = 1.000) and high separability for Rock (AUC = 0.971), Concrete (AUC = 0.93) and Wood (AUC = 0.942). This indicates strong generalization and robust discrimination under limited visual input. In contrast, models such as Swin (d) and ViT (c) exhibit reduced performance, particularly for the Rock and Wood categories, with AUC values dropping below 0.90. Despite moderate performance by Convolutional Autoencoder and MAE, their curves reflect less consistent margins across classes. SMARC’s curves, by comparison, are tighter and steeper, further confirming its classification confidence and reliability in recognizing materials from sparse visual cues.

#### 4.2.6. Qualitative Comparison

**Figure 4:**
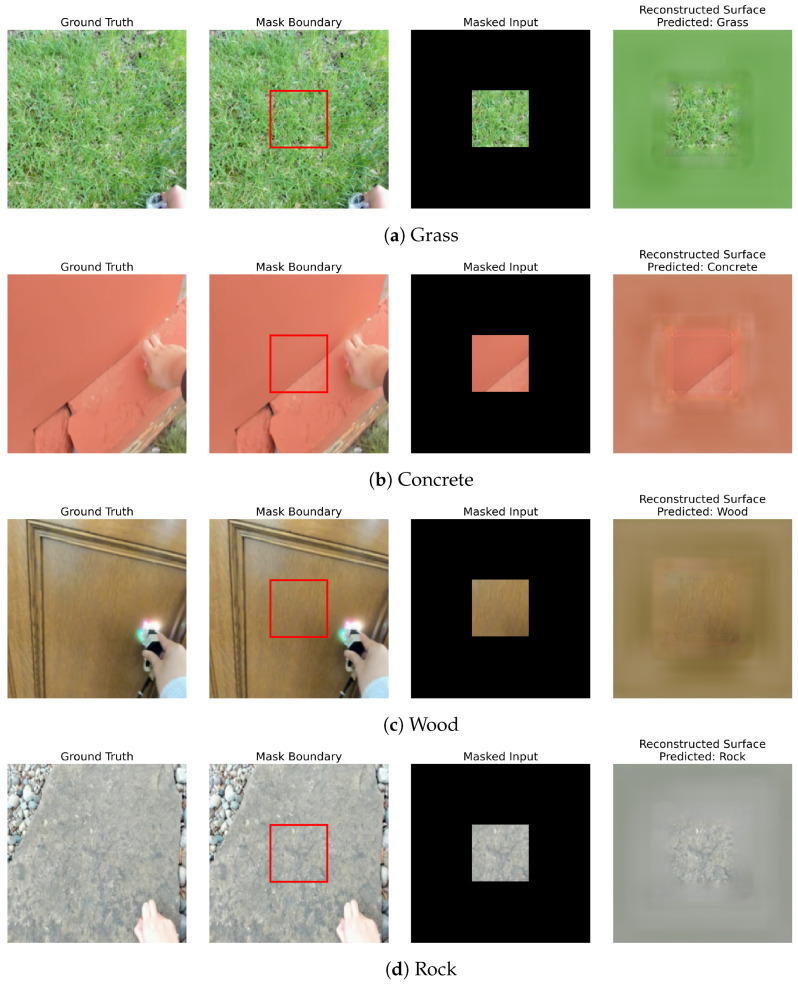
Qualitative reconstruction results under center continuous patch visibility. Each example shows the ground truth, mask boundary, the masked input, and the reconstructed surface with the predicted label.

Figure 4 illustrates the qualitative reconstruction results for four representative surface classes (grass, concrete, wood and rock) using the center continuous patch visibility setting. All the images are taken from the Touch and Go dataset throughout the experiment. For each case, the model is given only a centrally situated contiguous visible region, with the rest of the image entirely hidden.

Each row shows, from left to right, the ground truth surface, the mask border indicating the visible patch, the masked input and the reconstructed surface with the predicted material label. Despite the strong visibility constraint, SMARC reconstructs the global surface structure and preserves class-specific textural properties. These findings demonstrate the model’s potential capacity to execute spatial inpainting and semantic inference using highly localized visual signals. Although the overall structure and textural patterns are preserved, the reconstructions exhibit mild blurriness and smoothing artifacts. Fine-grained details and high-frequency texture variations are partially attenuated, indicating room for improvement in sharpness and structural continuity.

While qualitative data provide a visual representation of SMARC’s behavior, a comparative study clarifies how its design differs from existing approaches. Table 5 contrasts SMARC with prior reconstruction- and vision-based methods. While MAE, Uformer, Enhanced ViT, and PConv U-Net focus solely on pixel-level restoration or denoising, they lack multitask capability and semantic coupling. Surformer V1 performs classification but without reconstruction or masking. In contrast, SMARC integrates continuous patch masking with a joint reconstruction and classification objective, operating under only 10% visibility. This unified design enables both low-level recovery and high-level understanding, outperforming single-purpose baselines under low visibility conditions.

## 5. Conclusions

In this work, we introduced SMARC, a unified framework for Surface MAterial Reconstruction and Classification under extreme visual sparsity. Unlike traditional reconstruction or recognition pipelines that rely on dense visual input, SMARC learns to infer both texture and semantics from a single, continuous 10% patch of the image. By coupling a Partial Convolutional U-Net with a lightweight classification head, the model jointly performs spatial inpainting and material recognition within one coherent architecture. Extensive experiments on the Touch and Go dataset demonstrate SMARC’s ability to achieve high-fidelity reconstruction (17.55 dB PSNR) and robust material classification (85.10%), outperforming leading vision transformers and autoencoder baselines. Future extensions of SMARC will focus on improving reconstruction quality, classification accuracy and computational efficiency. We plan to explore parameter pruning, mixed precision training, and lightweight attention modules to reduce the model’s complexity and inference time while preserving visual quality. In addition, we aim to extend the evaluation of the proposed framework to other datasets with diverse surface materials. We are also exploring the development of a dedicated dataset for robotic surface perception, incorporating controlled sparse-view observations to further study reconstruction and classification under limited visual access.

## Figures and Tables

**Table 1:** Key intermediate variables in the SMARC architecture. Shapes are height × width × channels.

Variables	Shape	Description
S1_y, S2_y, S3_y, S4_y	224 × 224 × 64 → 28 × 28 × 512	Skip features from the four encoder blocks, providing multi-scale context to both the decoder and the classification head.
X1, X2, X3, X4	112 × 112 × 64 → 14 × 14 × 512	Downsampled encoder features (post pooling) passed to successive encoder stages, with X4 feeding the bottleneck.
S1_m, S2_m, S3_m, S4_m	224 × 224 × 1 → 28 × 28 × 1	Progressive binary masks propagated alongside skip features to preserve spatial validity for partial convolutions.
M1, M2, M3, M4	112 × 112 × 1 → 14 × 14 × 1	Downsampled masks aligned with X1–X4, with M4 passed to the bottleneck.
b, bm	14 × 14 × 2048, 14 × 14 × 1	Bottleneck feature and mask after dilated partial convolution blocks, providing global semantic context for reconstruction and classification.
y, m	224 × 224 × 64, 224 × 224 × 1	Final decoder feature and propagated mask prior to RGB output; masks are merged via element-wise max during upsampling.

**Table 2:** Reconstruction performance of all models on the Touch and Go dataset [22]. ↑ indicates higher is better and ↓ indicates lower is better. Best results are shown in bold, second-best are underlined, and third-best are shown in italic.

Model	PSNR ↑	SSIM ↑	MSE ↓	MAE ↓
Conv AE [5]	15.75	0.5458	0.0330	0.1382
MAE [11]	*17.01*	0.5265	*0.0267*	*0.1132*
ViT [9]	16.00	0.5211	0.0314	0.1342
Swin [10]	16.39	*0.5334*	0.0278	0.1221
DETR [21]	17.07	0.5289	0.0252	0.1115
**SMARC**	**17.55**	**0.5733**	**0.0223**	**0.0987**

**Table 3:** Classification performance comparison across all models. ↑ indicates higher is better. Best results are shown in bold, second-best are underlined, and third-best are shown in italic.

Model	Accuracy ↑	Precision ↑	Recall ↑	F1-Score ↑
Conv AE	0.8373	0.8371	0.8373	0.8362
MAE	*0.7466*	*0.7560*	*0.7466*	*0.7465*
ViT	0.7432	0.7503	0.7432	0.7443
Swin	0.7192	0.7366	0.7192	0.7232
DETR	0.7243	0.7351	0.7243	0.7253
**SMARC**	**0.8510**	**0.8568**	**0.8510**	**0.8514**

**Table 4:** Inference time comparison on the test dataset. Parameters/s denotes millions of parameters processed per second. Best results are shown in bold, second-best are underlined, and third-best are shown in italic.

Model	Total Parameters (Millions)	Total Time (s)	Inference Time/Img	Parameters/s
Conv AE	7.61	**2.98**	**0.0051**	2.53
MAE	35.28	*4.03*	*0.0069*	*8.75*
ViT	29.19	**2.98**	**0.0051**	9.68
Swin	**1.50**	6.66	0.0114	0.23
DETR	*7.87*	3.04	0.0052	2.59
SMARC	145.07	7.59	0.0130	**19.10**

**Table 5:** Qualitative comparison of existing models and the proposed approach. Our method supports joint reconstruction and classification under low visibility inputs on the Touch and Go dataset. ✓ indicates the presence of a feature, while ✗ indicates its absence [22].

Features	MAE [11]	Surformer -V1 [17]	Uformer [16]	Enhanced -ViT [9]	PConv U-Net [14]	SMARC (Ours)
Multitasking (reconstruction + classification)	✗	✗	✗	✗	✗	✓
Vision-based (RGB)	✓	✓	✓	✓	✓	✓
Semantic labeling compatible	✓	✓	✗	✗	✗	✓
Masked autoencoding	✓	✗	✗	✗	✗	✓
Explicit reconstruction	✗	✓	✓	✓	✓	✓
Masking strategy	random patches	none	none	rows occluded/noise	irregular (user defined)	continuous patch
Visible input ratio	25%	100%	100%	variable	variable	10%
Output dimensionality	2D image	class label	2D image	2D image	2D image	2D image + class label

## Data Availability

We used a publicly available dataset, which can be found at [22].

## Abbreviations

The following abbreviations are used in this manuscript:

AI	Artificial Intelligence
AUC	Area Under the Curve
CNN	Convolutional Neural Network
Conv AE	Convolutional Autoencoder
DETR	DEtection TRansformer
FC	Fully Connected Layer
GAP	Global Average Pooling
MAE	Masked Autoencoder
MAE (metric)	Mean Absolute Error
ML	Machine Learning
MSE	Mean Squared Error
PConv	Partial Convolution
PSNR	Peak Signal-to-Noise Ratio
ReLU	Rectified Linear Unit
RGB	Red, Green, Blue
ROC	Receiver Operating Characteristic
SE	Squeeze and Excitation
SMARC	Surface MAterial Reconstruction and Classification
SSIM	Structural Similarity Index Measure
Swin	Shifted Window Transformer
ViT	Vision Transformer
